# Total Alanine Aminotransferase (ALT) Flares in Pregnant North American Women With Chronic Hepatitis B Infection: Results From a Prospective Observational Study

**DOI:** 10.14309/ajg.0000000000000221

**Published:** 2019-08

**Authors:** Natalie H. Bzowej, Tram T. Tran, Ruosha Li, Steven H. Belle, Coleman I. Smith, Mandana Khalili, Ray Chung, Naoky Tsai, Norah Terrault

**Affiliations:** 1 Ochsner Medical Center, New Orleans, Louisiana, USA; 2 Cedar Sinai Medical Center, Los Angeles, California, USA; 3 University of Texas Health Science Center, Houston, Texas, USA; 4 University of Pittsburgh, Pittsburgh, Pennsylvania, USA; 5 Georgetown University Hospital, Washington, District of Columbia, USA; 6 University of California San Francisco, San Francisco, California, USA; 7 Massachusetts General Hospital, Boston, Massachusetts, USA; 8 University of Hawaii, Honolulu, Hawaii; 9 University of California, San Francisco, San Francisco, California, USA

## Abstract

**INTRODUCTION:**

Alterations in the immune system can result in alanine aminotransferase (ALT) flares either during pregnancy or after delivery in women with chronic hepatitis B virus (HBV) infection. The aim of this study was to prospectively assess changes in serum biochemical and virological markers of HBV infection during and after pregnancy in a large North American cohort of pregnant women with chronic HBV.

**METHODS:**

Adult pregnant women enrolled in the Hepatitis B Research Network between 2011 and 2016 were included. Serum ALT values and HBV DNA viral levels were obtained at <28 weeks and >28 weeks of gestation and <16 weeks, 16–31 weeks, and 32–48 weeks postpartum. Outcomes of ALT flares included severity, duration, and initiation of antiviral therapy.

**RESULTS:**

Amongthe 158 pregnant women with chronic HBV, the median age was 33 years, 73% were Asian, and 63% were hepatitis B e antigen (HBeAg) negative. The median HBV DNA level was substantially higher in the HBeAg-positive vs HBeAg-negative women (1.3 × 10^8^ vs 343 IU/mL), but serum ALT levels at their first study visit were similar. Among untreated pregnant women, there was a very mild increase in serum ALT postpartum among both HBeAg-positive and HBeAg-negative women *(P* < 0.001). Serum ALT flares (range 107–513 U/L) developed in 3.4% (5/149) during pregnancy and in 4.3% (4/92) after delivery. Twenty-two percent were initiated on antiviral therapy. After withdrawal of prophylactic anti-HBV therapy, 17.2% (5/29) developed serum ALT flares (range 107–208 U/L)within 14 weeks ofdrug discontinuation, and 3 additional women had flares despite continuous anti-HBV therapy during pregnancy or postpartum. Many ALT flares were not associated with significant changes in HBV DNA levels. No flares were severe with elevations of bilirubin or clinical decompensation.

**DISCUSSION:**

Spontaneous ALT flares in untreated pregnant women with chronic HBV are infrequent, mild, and self-limited both prepartum and postpartum. Although flares after the withdrawal of antiviral therapy postpartum are more common, they were also mild and self-limited. Further studies of the immunopathogenesis of pregnancy-related flares are needed, as well as effects on long-term outcome of the mother and infant.

## INTRODUCTION

Pregnancy and the postpartum period are associated with unique changes in the immune system that may impact the natural history of autoimmune diseases and immune-mediated infections (1). Although pregnant women with chronic hepatitis B virus (HBV) have maternal and fetal outcomes comparable to uninfected women, a minority of women may experience alanine aminotransferase (ALT) flares, i.e., increases in serum ALT above the upper limits of normal (ULN) either during pregnancy or after delivery. The frequency and severity of flares reported in the literature are highly variable. Among untreated pregnant women with chronic HBV, the reported incidence of ALT flares varies between 0.3% and 9% (2–4). Although spontaneous flares were not associated with clinical decompensation in most patients, severe flares necessitating liver transplantation when salvage antiviral therapy failed have been rarely reported (4–6).

In the postpartum period, ALT flares have been reported in 25%–44.7% of untreated women (7–12) and were more likely to occur in hepatitis B e antigen (HBeAg)-positive patients (9). Flares in these studies generally occurred within 3 months of delivery, were asymptomatic, and resolved spontaneously. It has been postulated that postpartum ALT flares may arise to rapid immune restitution against HBV antigens in the liver (7,8). The impact of antiviral therapy during pregnancy and withdrawal after delivery may also predispose to flares because HBV flares induced by the withdrawal of antiviral therapy have been well described outside of pregnancy (13–15). An increasing proportion of pregnant women with high viral loads are now receiving prophylactic oral antivirals to reduce the risk of HBV transmission to their offspring (16). In these instances, the drug is discontinued in the postpartum period to facilitate breastfeeding and to minimize the risk of drug-resistant HBV. The impact of drug discontinuation in those receiving prophylactic antivirals has not been well studied. Overall, prospective, longitudinal data in large numbers of women describing the natural history of hepatitis B during and after pregnancy are lacking. We therefore investigated the frequency and clinical and virological characteristics of serum ALT flares during pregnancy and postpartum in a large, racially diverse North American cohort of women with chronic hepatitis B.

## PATIENTS AND METHODS

The Hepatitis B Research Network (HBRN), sponsored by the National Institute ofDiabetes and Digestive andKidneyDiseases, comprises 20 adult liver centers in the United States and 1 center in Toronto, Canada (17). The HBRN enrolled HBsAg-positive persons aged at least 18 years who did not have a history of hepatic decompensation, hepatocellular carcinoma, transplantation, or HIV coinfection. For the current study, all women enrolled into the HBRN who were either pregnant at the time of enrollment or became pregnant after enrollment were included. Decisions related to initiation or discontinuation of antiviral therapy were made by treating physicians on a clinical basis and not protocol mandated. As part of the HBRN adult cohort study, all participants had demographic and clinical data collected at enrollment, week 12, week 24, and then every 24 weeks. Per protocol, pregnant participants were evaluated twice during pregnancy, at <28 weeks (unless they were enrolled at a later stage in their pregnancy) and at >28 weeks of gestation, and 3 times postpartum, at <16 weeks, at 16–31 weeks, and at 32–48 weeks. Participants experiencing hepatitis flares were seen at more frequent intervals. For additional pregnancy visits, biochemical (liver biochemistries) and virological (HBV DNA level, HBeAg, and anti-HBe) tests performed as part of standard of care were collected. At each pregnancy and postpartum visit, use of antiviral treatment was captured. At the first postpartum visit, information regarding pregnancy viability and method of delivery was collected. We acknowledge the importance of screening all infants born to HBV-infected mothers for evidence of HBV infection and regret that the logistics of this study did not allow us to collect these data.

For these analyses, an ALT flare was defined *a priori* as a transient rise of serum ALT up greater than ≥5 times the ULN (20 U/L). Flares were graded as either mild (rise of ALT to ≥5 times ULN but <10times ULN) or moderate (ALT rise to ≥10 times ULN). Flares were considered severe if jaundice was present (bilirubin ≥2.5 mg/dL at the time of the flare) or if there were of symptoms suggestive of liver injury. Each flare was reviewed by the HBRN Adjudication Committee to confirm that the etiology of the flare was pregnancy related and not due to alcohol, drug, gallstones, or other causes of liver injury. The duration of ALT flare was calculated by week of first qualifying ALT elevation to week of fall of ALT into the normal range (<20 U/L or preflare level). Flare type was characterized as (i) spontaneous (if ALT rise occurred without antiviral therapy during or after pregnancy), (ii) on therapy (if ALT rise occurred on treatment during pregnancy or postpartum), or (iii) after withdrawal of therapy (if ALT rise occurred within 24 weeks of discontinuation of antiviral therapy). A rise in HBV DNA with a flare was defined as at least a 10-fold increase from the preflare value.

All protocols were approved by the HBRN Steering Committee and the institutional review boards (Research Ethics Board in the case of the Toronto site) of the participating sites, and all participants provided written informed consent. This analysis is part of the Hepatitis B Research Network Adult Cohort Study and is registered in ClinicalTrials.gov (NCT01263587).

### Statistical analysis

Data are summarized via frequencies and percentages for categorical variables and via median and range for continuous variables.

To estimate changes in serum ALT and HBV DNA over time, mixed effects models during gestation and postpartum with data collected after antiviral therapy were initiated or the next gestation excluded. Subjects with outcomes of miscarriage or termination were also excluded. The mean of log_10_ ALT (U/L) in a linear mixed effects model was estimated and then transformed back to a geometric mean of ALT (U/L) through 10× transformation. The covariates in the mixed model include pregnancy stage, HBeAg, pregnancy interval by HBeAg interaction, and log_10_-transformed HBV DNA (log_10_ IU/mL). Similarly, we modeled log_10_-transformed HBV DNA using a linear mixed effects model that includes pregnancy stage, HBeAg, and their interaction.

We summarized the frequency of flares during different types of follow-up periods to account for the change in pregnancy stage (during gestation and postpartum) and antiviral treatment status (untreated, on treatment, and after treatment withdrawal). Besides the raw percentage of flares, we also estimated the incidence rate (IR) using Poisson regression, accounting for the total length of follow-up (in person-months) during different types of periods.

## RESULTS

**Figure 1.**
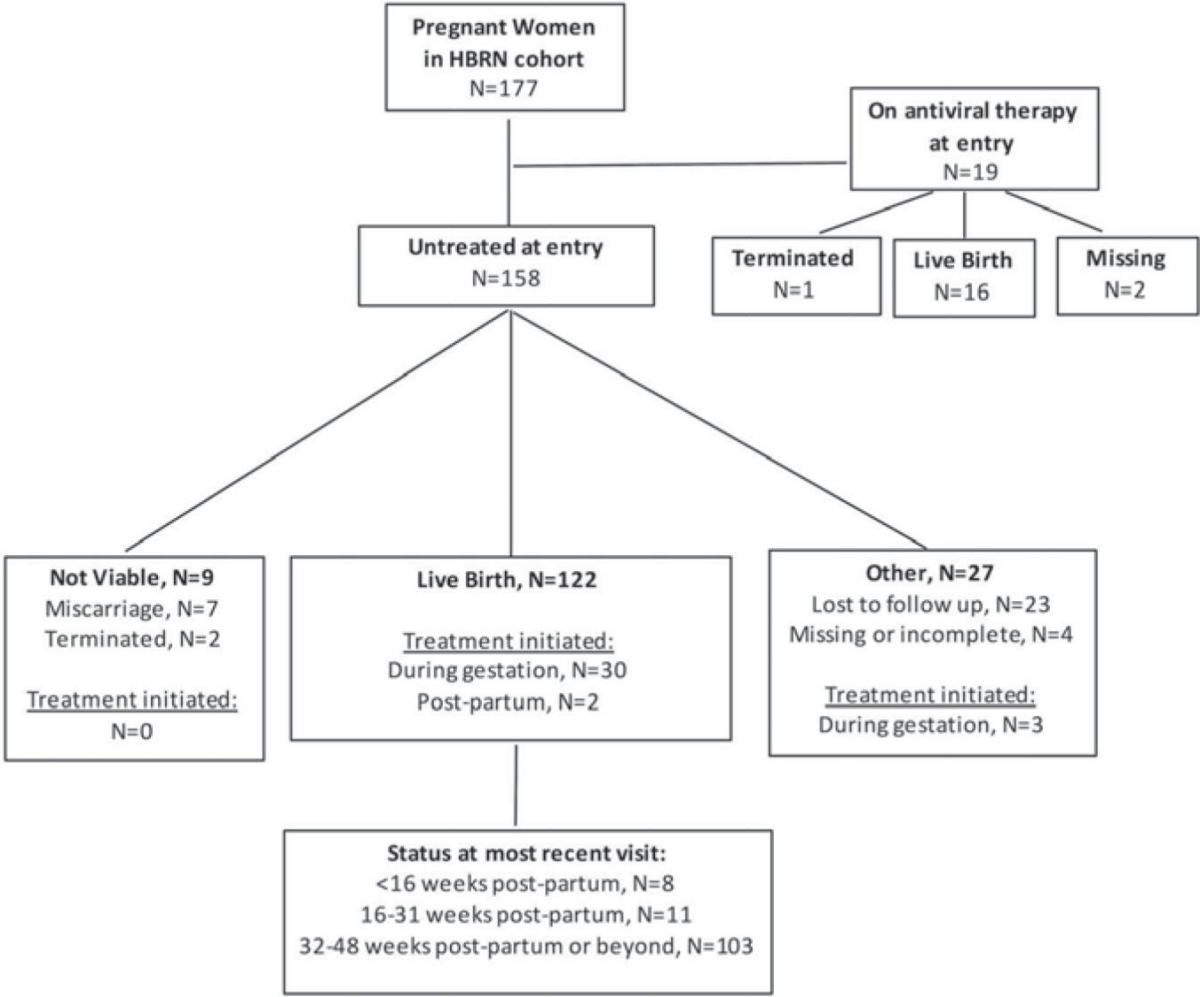
Summary of analytic sample, pregnancy outcome, and antiviral treatment. HBRN, Hepatitis B Research Network.

A total of 177 women with chronic HBV in the HBRN were enrolled during pregnancy or became pregnant after enrollment between January 2011 and May 2016 (Figure 1). Nineteen were on treatment at their first pregnancy visit and were excluded from the analysis of the natural history of HBV during pregnancy and after delivery but included in the analysis of flares. Characteristics of the women at their first pregnancy visit are shown in Table 1. The median age was 33 years (range 18–51 years). The majority of pregnant women were Asian (73%), followed by black (18%), white (8%), and other race (1%). Only aminority (16%) ofwomen were born in North America. At the first pregnancy visit, 63% of women were negative for HBeAg. Thirty-one percent had an HBV DNA level greater than 200,000 IU/mL, and 50% had an HBV DNA level less than 1,000 IU/mL. The most common genotypes were B and C (38% and 33%, respectively). Phenotype distribution was similar to that of the overall HBRN cohort (18): 10% were immune tolerant, 20% immune active (HBeAg positive), 4% immune active (HBeAg negative), 27% inactive carriers, and 39% indeterminant.

## PREGNANCY OUTCOMES

Pregnancy outcomes were available for 131 (83%) of the 158 participants (Figure 1), with 122 (93%) resulting in live births. The 9 nonviable pregnancies (7%) were due to 7 miscarriages and 2 terminated pregnancies. None of the women who experienced miscarriages experienced flares or were on antiviral treatment for hepatitis B during pregnancy. The mode of delivery was vaginal birth in 76% (93/122) and C-section in 24% (29/122).

## SERUM ALT AND HBV DNA LEVELS DURING PREGNANCY AND POSTPARTUM

**Figure 2.**
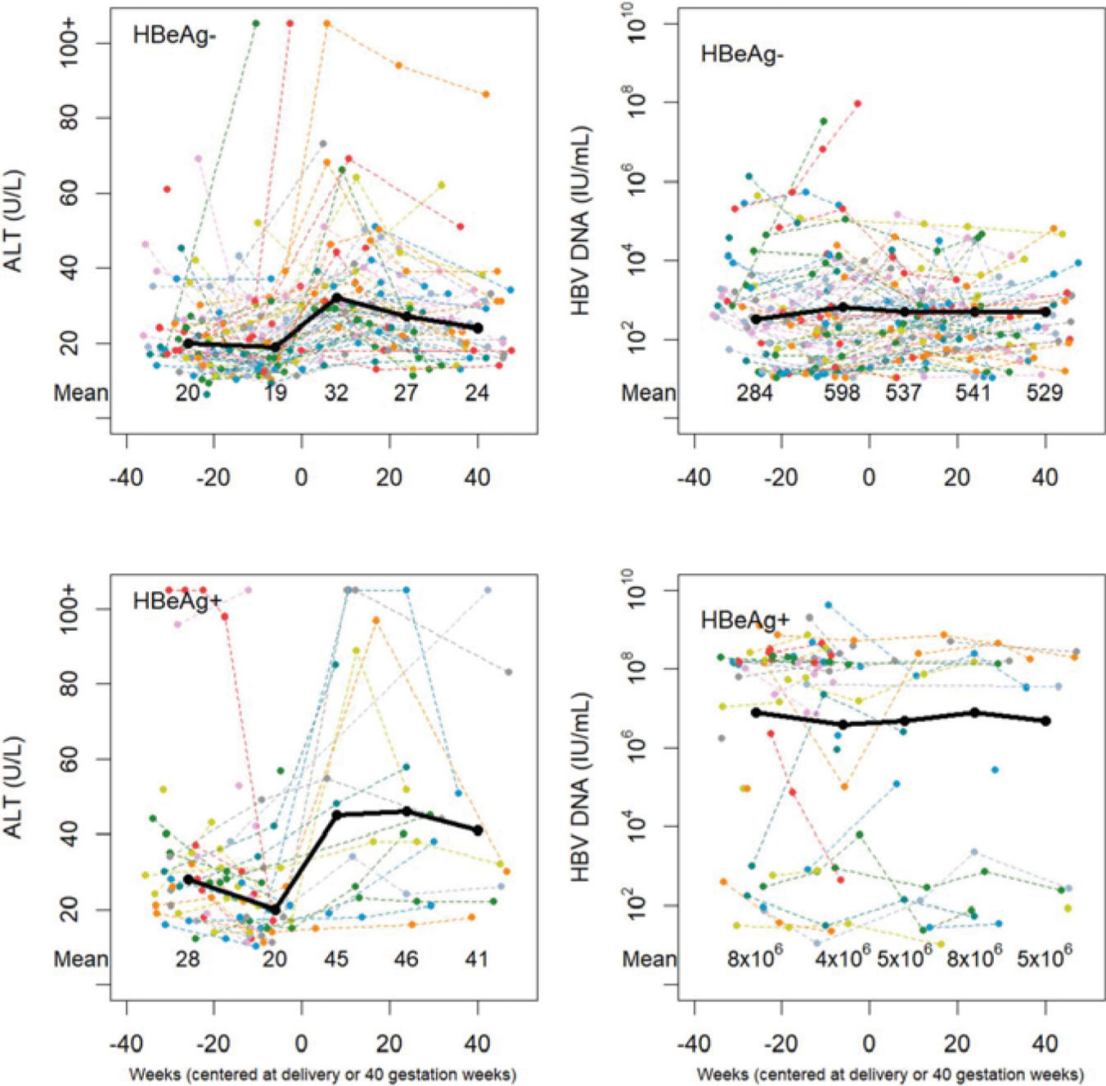
Scatterplots of ALT and HBV DNA vs time during pregnancy and postpartum. ALT, alanine aminotransferase; HBV, hepatitis B virus.

The mean serum ALT did not change significantly during gestation in HBeAg-negative patients (Figure 2), whereas there was a very mild decrease among HBeAg-positive patients in the third trimester compared with the first 2 trimesters (*P =* 0.001). Moreover, there was a mild increase in serum ALT postpartum among both HBeAg-positive and HBeAg-negative women (*P* < 0.001). In contrast, HBV DNA levels did not change significantly over time for HBeAg-negative women (*P* = 0.14) nor HBeAg-positive women (*P* = 0.68) (Figure 2).

## USE OF ANTIVIRAL THERAPY DURING PREGNANCY

Of 158 pregnant women, 35 (22%) were initiated on antiviral treatment during gestation (N = 33) or postpartum (N = 2). Nearly all who began antiviral therapy during gestation were HBeAg positive (30/32,94%; 1 missing), and 29/32 (91%) had HBV DNA ≥200,000 IU/mL. The median gestation week when treatment was started was 29 weeks (range 16–39 weeks). The percentage of women receiving each antiviral was as follows: tenofovir 76%, lamivudine 21%, and lamivudine switched to tenofovir 3%. Treatment was discontinued (the reasons for discontinuation were unknown) in 24: within 7 days of delivery for 13, between 1 and 12 weeks for 9, and between 32 and 48 weeks after delivery for 2. Treatment was continued in 4, and data for 5 were missing.

## SERUM ALT FLARES

Table 2 summarizes the women with mild to moderate flares during different periods (gestation, postpartum, and after antiviral withdrawal). The baseline characteristics of these corresponding subgroups are provided in Table S1 (Supplementary Digital Content 1, http://links.lww.com/AJG/A167). Of the 177 women, a total of 16 women ever flared during the follow-up. One woman experienced 2 flares. Pregnancy outcomes were recorded for 15, and all were live births. The rates and IRs of flares were low, with higher values observed after the withdrawal of antiviral therapy (Table 2).

In analyses stratified by HBeAg, we observe a lower rate and IR of flares among HBeAg-negative women compared with HBeAg-positive women (Table 2). Among some HBeAg-positive women, flares occur on antiviral therapy. The IR of flares on antiviral therapy was slightly lower than their counterparts during untreated periods, although the low incidence of flares precluded formal testing. Detailed information on the women with flares is reported below.

### Spontaneous ALT flares

Among untreated women, ALT flares were observed in 3.4% (5/149) during pregnancy and 4.3% (4/92) during postpartum (Table 2 and Figure S1, Supplementary Digital Content 1, http://links.lww.com/AJG/A167). The IRof flares was 0.009 per person-month during gestation and 0.004 per person-month postpartum. Of the 5 flares during pregnancy, 4 were moderate (ALT range 222–513), occurred close to or during the third trimester (27–39 gestationweeks), andwere startedon antiviraltherapy. Of the 4 flares in the postpartum period, 3 occurred within the first 3 months, all but 1 were mild flares (ALT range 107–129), and only 1 postpartum flare (mild) was started on antiviral therapy. Duration of flares varied from 15 weeks to >48 weeks.

Median HBV DNA among those who experienced flares was 3.5 × 10^7^ IU/mL (range 3.9 × 10^4^ to 5.2 × 10^8^ IU/mL). Of the 6 HBeAg-positive women with flares, HBV DNA decreased by more than 10-fold for 3 and remained stably high for 2 (1 missing). Only 1 flare was associated with a transient loss of HBeAg. In univariable analysis, associating spontaneous flares with baseline characteristics, only higher HBV DNA attained statistical significance (*P =* 0.005) (see Table S2 in Supplemental Appendix, Supplementary Digital Content 1, http://links.lww.com/AJG/A167). The percentage of patients with positive HBeAg was numerically higher in the flare group (67% vs 35%), but the difference was not statistically significant (*P* = 0.074).

### Serum ALT flares on antiviral therapy

An ALT flare on antiviral therapy was observed in 2% (1/49) during gestation and 5.6% (2/36) postpartum (Table 2 and Figure S2, Supplementary Digital Content 1, http://links.lww.com/AJG/A167). In all patients, antiviral therapy was continued and the flare resolved. In the 2 women with postpartum flares, antiviral therapy had been started in the third trimester, and the flares occurred at 7 and 13 weeks postpartum. HBV DNA levels were elevated (range 6,980–7.4 × 10^5^ IU/mL) at the time of the flare and declined postflare in both women. No HBeAg loss was observed.

### Serum ALT flares after withdrawal of antiviral therapy

Of all 177 women, discontinuation of antiviral therapy was observed among 30 (Table 2 and Figure S3, Supplementary Digital Content 1, http://links.lww.com/AJG/A167). The majority of the discontinuations occurred either within 7 days of delivery (50%) or between 1 and 12 weeks postpartum (33%). Twenty-nine had follow-up data after the withdrawal of therapy, and an ALT flare was observed in 17.2% (5/29). The IR of flare was 0.020 per person-month. Four flares were mild (ALT range 107–199 U/L), and 1 was moderate (ALT 208 U/L). HBV DNA levels ranged from 2.2 × 10^4^ to 3.5 × 10^8^ IU/mL at the time of the flare, with an increase noted in 2 and decrease in 1. Flares occurred 7–14 weeks after discontinuation of treatment andwere prolonged (16 to >26 weeks). ALT levels improved without reinitiation of therapy, and 1 woman lost HBeAg.

## DISCUSSION

This represents one of the few prospective studies of pregnant women with chronic HBV infection and the only study of North American women. Reflecting the population of CHB in the United States and Canada, women were predominantly foreign born and of Asian or African race. Importantly, the vast majority were untreated during pregnancy and postpartum, providing a unique opportunity to evaluate the effect of pregnancy on the natural history of CHB.

Pregnancy has been viewed as a period of immunological tolerance with relative immune quiescence, followed by functional immune reconstitution (20). Indeed, a statistically significant but clinically mild increase in ALT levels was observed early after delivery, suggesting increase immune activity, without significant changes in HBV DNA or seroconversion events. The increase in ALT early after delivery in this study was observed in both HBeAg-negative and HBeAg-positive women and is consistent with previous reports (10). We also observed a very mild decline in ALT in the third trimester compared with the first 2 trimesters among HBeAg-positive women. Similar to other studies (10) reporting changes in ALT during pregnancy, the magnitude of change was very modest and clinically insignificant. Of greater interest and potential consequence is the frequency of ALT flares. In our prospective study, the overall rate of ALT flares during pregnancy was 3.4% and postpartum was 4.3%, but the rates were influenced by the use of antiviral therapy.

Among untreated women, spontaneous ALT flares occurred in an approximately equal distribution during pregnancy and in the postpartum period. Although the incidence was low, the majority of flares were mild (>5 times the ULN) or moderate (> 10 times ULN) in intensity but without bilirubin elevation and many resolved without need for antiviral therapy. Interestingly, there was no significant change in HBV DNA apparent with some of the flares, although we acknowledge the possibility that changes in HBV DNA may have been missed, given the frequency of monitoring. In terms of important immunologic end points, only 1 patient had a transient loss of HBeAg in the context of a mild flare. Overall, our results are reassuring, both in terms of the frequency of flares and their outcomes without treatment. The rate of spontaneous ALT flares in other retrospective studies varies from 9% to 25%, with higher rates reported postpartum than during pregnancy (3–5,10), and the variability in rates is largely related to how flares were defined. Rare, severe outcomes have been reported in retrospective studies, but because of the lack of details on baseline liver disease, severity, and other factors that may cause ALT elevation, it is difficult to assign causality to HBV. In our study, none of the women had advanced fibrosis based on clinician assessment and AST-to-platelet index ratio.

There is no generally accepted universal definition of a significant increase in liver disease activity or “flare” during or after pregnancy. We used 5 times the ULN of ALT to define mild flares and 10 times the ULN to define moderate flares. Recent studies have used much more liberal definitions for ALT flares: any ALT > ULN (normal ALT = 40); an ALT > 2 times the ULN or twice the upper limit of baseline ALT (whichever is higher); and ALT >5 times the ULN or >3 times ULN (whichever is higher) (9,11,19). These differing definitions of ALT flares make comparisons between studies difficult. We chose the higher cutoffs of ALT to define flares because these were felt to be more clinically relevant, more likely to lead to change in clinical management (i.e., addition of antiviral therapy), and potentially more likely to be associated with seroconversion events. Thus, the higher ALT cutoff used for flare in our study likely accounts for the lower IR. Alternatively, this could be related to the frequency of visits because visit windows were deliberately generous to maximize patient retention in this observational study. Women were seen twice during pregnancy and 3 times after delivery (<16 weeks, 16–31 weeks, and 32–48 weeks). Postpartum flares have been found to commonly occur within the first 12 weeks postpartum and resolve spontaneously (9). Three-month evaluations after delivery have been used in other studies (19). Thus, it is possible that with the lack of symptoms during flares and the visit schedule in this study, flares could have been missed.

ALT flares on antiviral therapy occurred in 2% during pregnancy and 5.6% postpartum, a frequency not dissimilar to women not on treatment. These 3 flares (2 postpartum and 1 in second trimester) resolved without interruption or change in antiviral treatment. Information on adherence was not specifically captured, but in 2 of 3 women, the HBV DNA levels were low or declined during the flares, suggesting that nonadherence was not an issue. None of these flares were associated with seroconversion. In the limited other reports on ALT flares during antiviral therapy, similar rates are seen. For example, in a randomized clinical trial of telbivudine started at weeks 26–28 of pregnancy, 8% experienced ALT flare (defined as ALT >2 × ULN) (20). Although the ALT flares did not result in decompensated liver disease, they occurred throughout pregnancy and after delivery. Moreover, although flares were observed rarely in those on continued anti-HBV therapy, this finding suggests that treatment may not prevent flares in all cases and is consistent with previously published reports (11,20).

The highest rate of ALT flares was seen in women who discontinued antivirals during pregnancy or after delivery. In total, 17.2% of women experienced a mild to moderate flare within 14 weeks of stopping antivirals. Only 1 seroconversion event occurred in association with these flares, and all resolved without need for reinstitution of antiviral therapy. Reported rates of flare after withdrawal of antiviral therapy in women who discontinued therapy shortly after delivery have been highly variable (5%–62%) (8,10–12,19–23), potentially related to the intensity of ALT measurements (monthly vs every 3 months), rapidity and/or magnitude of HBV DNA increases with treatment discontinuation, or the duration of antiviral therapy before discontinuation. Our findings are consistent with a recent randomized clinical trial in which women receiving tenofovir during late pregnancy to early postpartum had significantly higher rates of ALT elevation than untreated women (45% vs 30%) (12). To date, predictors of these postpartum treatment flares remain elusive, but most women remain off treatment. Regardless, this highlights the importance of monitoring all women after delivery to determine whether there is any reason to consider reinstitution of antiviral therapy.

This study has a number of limitations. First, most women did not become pregnant after enrollment into the HBRN; rather, they were already pregnant at enrollment. Thus, many women did not have data before pregnancy for more robust baseline comparison. In addition, if women were recruited into the study already pregnant, it is not known whether they discontinued treatment before cohort enrollment. Prepregnancy treatment could influence rates of flare during pregnancy and was observed to be 16% in a recent study (19). Furthermore, in this study, no one discontinued antiviral therapy in the first or second trimester of pregnancy. Another limitation is missing laboratory results because laboratory tests were performed as part of standard of care and not mandated by the study protocol and lack of standardized protocol on timing or type of antiviral therapy. Because the number of subjects in each phase of hepatitis B was relatively small, this study may not have captured differences in the rate of flare between subgroups. In addition, we did not evaluate rates of mother-to-child transmission and thus could not assess the effect of flares and antiviral treatment on perinatal transmission. However, this was a multicenter prospective observational study that included patients from different practice types and different regions in the United States and Canada with varying ethnicity and HBV genotypes.

In conclusion, ALT levels remain stable during pregnancy in most patients with chronic HBV, but mild increases were observed early after delivery, without concomitant changes in HBV DNA. Although ALT flares occurred in 3.4% during pregnancy and 4.3% postpartum among untreated women and in 17% of those who stopped antiviral therapy, initiation of antiviral therapy was rarely required, and no clinically significant decompensating events occurred. Although total published experience with ALT flares during antiviral therapy is still quite limited, the available data suggest that continuing treatment is the appropriate strategy.

## Supplementary Material

AJG_2019_03_06_BZOWEJ_AJG-18-2076_SDC1

## Figures and Tables

**Table 1.** Baseline data from the first pregnancy visit of women enrolled in the HBRN

Feature	Number	Proportion or median (range)
Age (yr)	158	33 (18–51)
Race		
White	13	8%
Black	29	18%
Asian	115	73%
Other	1	1%
Born in North America	26	16%
Serum ALT (U/L)	153	22 (5–329)
HBeAg positive	55	25 (12–329)
HBeAg negative	92	21 (6–69)
HBV DNA (IU/mL)	156	978 (BLQ, ALQ)
Among HBeAg positive	56	1.3 × 10^8^ (1.5, ALQ)
Among HBeAg negative	94	343 (BLQ, 6.1)
HBV DNA (IU/mL)	156	
<1,000	78	50%
1,000–200,000	30	19%
>200,000	48	31%
HBeAg	151	
Positive	56	37%
Negative	95	63%
APRI	129	0.3 (0.1, 0.8)
Genotype	138	
A	25	18%
B	52	38%
C	46	33%
D	8	6%
Other (E and F)	7	5%
Phenotype (18)	146	
IT, HBeAg positive	15	10%
IA, HBeAg positive	29	20%
IA, HBeAg negative	6	4%
IC, HBeAg negative	39	27%
Indeterminant	57	39%

BLQ ≤ 20 IU/mL; ALQ ≤ 170,000,000 IU/mL.

ALQ, above limits of quantitation; ALT, alanine aminotransferase; APRI, AST-to-platelet ratio index; HBeAg, hepatitis B e antigen; BLQ, below limits of quantitation; HBRN, Hepatitis B Research Network; HBV, hepatitis B virus; IA, immune active; IC, inactive carrier; IT, immune tolerant.

**Table 2.** Women with mild to moderate flares during different types of follow-up periods, overall, and by baseline HBeAg

Type of follow-up period	No. of women with follow-up^a^	Total follow-up (person mo)	Mild or moderate flares (ALT ≥ 100 U/L)		
			Frequency^b^	%	Incidence rate (per person per mo)
Overall					
Untreated					
During gestation	149	529	5	3.4	0.009
Postpartum	92	891	4	4.3	0.004
On treatment					
During gestation	49	111	1	2.0	0.009
Postpartum	36	207	2	5.6	0.010
After wlthdrawal^c^	29	249	5	17.2	0.020
HBeAg+					
Untreated					
During gestation	56	171	3	5.4	0.018
Postpartum	19	190	3	15.8	0.016
On treatment					
During gestation	40	81	1	2.5	0.012
Postpartum	30	141	2	6.7	0.014
After withdrawal	26	230	5	19.2	0.022
HBeAg−					
Untreated					
During gestation	87	344	2	2.3	0.006
Postpartum	71	680	1	1.4	0.001
On treatment					
During gestation	6	22	0	0	—
Postpartum	5	54	0	0	—
After withdrawal	1	11	0	0	—

ALT, alanine aminotransferase; HBeAg, hepatitis B e antigen.

a All 177 pregnant women were considered. Women with nonviable outcomes were excluded.

b Total of 17 flares in 16 women; 1 woman experienced 2 flares.

c Combining both during gestation and postpartum.

## Study Highlights

### WHAT IS KNOWN

- Although pregnant women with chronic HBV have maternal and fetal outcomes comparable to uninfected women, women may experience ALT flares either during pregnancy or after delivery.
- Antiviral therapy during pregnancy and withdrawal after delivery may predispose to flares.
- Prospective, longitudinal data in large numbers of women describing the natural history of hepatitis B during and after pregnancy are lacking.

### WHAT IS NEW HERE

- Among untreated pregnant women, there was a very mild increase in serum ALT postpartum.
- Serum ALT flares developed in 3.4% during pregnancy and in 4.3% after delivery.
- After withdrawal of prophylactic anti-HBV therapy, 17.2% developed serum ALT flares within 14 weeks of drug discontinuation.
- Flares occurred despite continuous anti-HBV therapy during pregnancy or postpartum.
- Many ALT flares were not associated with significant changes in HBV DNA levels.
- No flares were severe—there were no elevations of bilirubin or clinical decompensation.
