# Supplementary material for: Total Alanine Aminotransferase (ALT) Flares in Pregnant North American Women With Chronic Hepatitis B Infection: Results From a Prospective Observational Study
Source: Am J Gastroenterol. Author manuscript; Available in PMC 2020 Aug 1. (PMC7132838; doi:10.14309/ajg.0000000000000221)
Supplement: AJG_2019_03_06_BZOWEJ_AJG-18-2076_SDC1 [file NIHMS1564031-supplement-AJG_2019_03_06_BZOWEJ_AJG-18-2076_SDC1.docx]

**Supplementary Appendix**

**Table S1**: Baseline characteristics of participants who were followed during different types of periods. The subgroups are not mutually exclusive, as the same woman may undergo different types of periods.

|  | **Subgroup1**  **Untreated Gestation (N=149)*** | | **Subgroup2**  **Untreated Postpartum (N=92)** | | **Subgroup3**  **On treatment Gestation (N=49)** | | **Subgroup4**  **On treatment Postpartum (N=36)** | | **Subgroup5**  **After**  **withdrawal (N=29)** | |
| --- | --- | --- | --- | --- | --- | --- | --- | --- | --- | --- |
|  | **N** | **% or**  **Median(range)** | **N** | **% or**  **Median(range)** | **N** | **% or**  **Median(range)** | **N** | **% or**  **Median(range)** | **N** | **% or**  **Median(range)** |
| **Age (years)** | 149 | 32(18,49) | 92 | 33(22,51) | 49 | 31(23,40) | 36 | 31(23,40) | 29 | 31(24,40) |
| **Race** |  |  |  |  |  |  |  |  |  |  |
| White | 12 | 8% | 10 | 11% | 1 | 2% | 1 | 3% | 0 | 0% |
| Black | 27 | 18% | 19 | 21% | 3 | 6% | 2 | 6% | 1 | 3% |
| Asian | 109 | 73% | 62 | 67% | 43 | 88% | 31 | 86% | 28 | 97% |
| Other | 1 | 1% | 1 | 1% | 2 | 4% | 2 | 6% | 0 | 0% |
| **Genotype** |  |  |  |  |  |  |  |  |  |  |
| A | 22 | 17% | 17 | 22% | 1 | 2% | 0 | 0% | 0 | 0% |
| B | 49 | 38% | 32 | 41% | 16 | 33% | 12 | 34% | 11 | 38% |
| C | 45 | 35% | 20 | 25% | 27 | 56% | 20 | 57% | 16 | 55% |
| D | 6 | 5% | 7 | 9% | 2 | 4% | 1 | 3% | 1 | 3% |
| Other | 7 | 5% | 3 | 4% | 2 | 4% | 2 | 6% | 1 | 3% |
| **Born in North America** | 26 | 17% | 17 | 18% | 4 | 8% | 3 | 8% | 3 | 10% |
| **HBeAg** |  |  |  |  |  |  |  |  |  |  |
| Positive | 56 | 39% | 19 | 21% | 40 | 87% | 30 | 86% | 26 | 96% |
| Negative | 87 | 61% | 71 | 79% | 6 | 13% | 5 | 14% | 1 | 4% |
| **ALT (U/L)** |  |  |  |  |  |  |  |  |  |  |
| Overall | 144 | 22(5,513) | 88 | 21(6,69) | 49 | 25(9,513) | 36 | 25(9,513) | 29 | 20(12,135) |
| HBeAg+ | 55 | 25(12,513) | 18 | 25(12,44) | 40 | 25(9,513) | 30 | 25(9,513) | 26 | 20(12,135) |
| HBeAg- | 84 | 21(6,69) | 68 | 21(6,69) | 6 | 21(11,70) | 5 | 25(11,70) | 1 | 17 |
| **HBV DNA (IU/mL)** | | |  | |  |  |  |  |  |  |
| Overall | 147 | 1208(BLQ,ALQ) | 92 | 362(BLQ,4.1×10^9^) | 49 | 7.3×10^6^(BLQ,ALQ) | 36 | 9.5×10^6^(BLQ,ALQ) | 29 | 1.2×10^8^(BLQ,ALQ) |
| HBeAg+ | 56 | 1.3×10^8^(31,ALQ) | 19 | 4.1×10^7^(31, 4.1×10^9^) | 40 | 5.2×10^7^(BLQ,ALQ) | 30 | 4.3×10^7^(BLQ,ALQ) | 26 | 1.3×10^8^(BLQ,ALQ) |
| HBeAg- | 86 | 350(BLQ,1.3×10^6^) | 71 | 210(BLQ, 4.2×10^5^) | 6 | 465(BLQ,2.0×10^5^) | 5 | 258(BLQ,2.0×10^5^) | 1 | 672 |
| **APRI** |  |  |  |  |  |  |  |  |  |  |
| Overall | 124 | 0.3(0.1,4.4) | 77 | 0.3(0.1,0.8) | 42 | 0.3(0.2,4.4 | 32 | 0.3(0.2,4.4) | 26 | 0.3(0.2,0.8) |
| HBeAg+ | 47 | 0.3(0.1,4.4) | 15 | 0.2(0.1,0.7) | 35 | 0.3(0.2,4.4) | 27 | 0.3(0.2,4.4) | 24 | 0.3(0.2,0.8) |
| HBeAg- | 74 | 0.3(0.1,0.8) | 61 | 0.3(0.1,0.8) | 5 | 0.5(0.2,0.6) | 4 | 0.5(0.2,0.6) | 1 | 0.4 |
| **Phenotype** |  |  |  |  |  |  |  |  |  |  |
| IT, HBeAg+ | 15 | 11% | 3 | 3% | 15 | 33% | 11 | 31% | 13 | 48% |
| IA, HBeAg+ | 28 | 20% | 7 | 8% | 16 | 35% | 12 | 34% | 11 | 41% |
| IA, HBeAg- | 6 | 4% | 4 | 5% | 0 | 0% | 0 | 0% | 0 | 0% |
| IC, HBeAg- | 36 | 26% | 30 | 35% | 2 | 4% | 1 | 3% | 1 | 4% |
| Indeterminant | 53 | 38% | 42 | 49% | 13 | 28% | 11 | 31% | 2 | 7% |

*All but one participant in subgroup 1 were untreated at enrollment. For that participant, antiviral therapy initiated 6 days before enrollment. As this participant was an adjudicated spontaneous flare based on clinical notes, we included her in the denominator for calculating spontaneous flare rates.

**Table S2:** Baseline characteristics among women who experienced a spontaneous flare (N=9) vs. women who did not experience any flare during the follow-up (N=136).

|  | **Spontaneous flare** | | **No Flare*** | | **p-value^&^** |
| --- | --- | --- | --- | --- | --- |
|  | **Number** | **Proportion or median (range)** | **Number** | **Proportion or median (range)** |  |
| **Age (years)** | 9 | 31(27,37) | 136 | 32(18,51) | 0.649 |
| **Race** |  |  |  |  | 0.377 |
| White | 1 | 11% | 11 | 8% |  |
| Black | 0 | 0% | 27 | 20% |  |
| Asian | 8 | 89% | 97 | 71% |  |
| Other | 0 | 0% | 1 | 1% |  |
| **Born in North America** | 1 | 11% | 25 | 18% | >0.9 |
| **Genotype** |  |  |  |  | 0.497 |
| A | 0 | 0% | 22 | 19% |  |
| B | 4 | 44% | 45 | 39% |  |
| C | 4 | 44% | 37 | 32% |  |
| D | 1 | 11% | 6 | 5% |  |
| Other (E & F) | 0 | 0% | 6 | 5% |  |
| **HBeAg** |  |  |  |  |  |
| Positive | 6 | 67% | 45 | 35% | 0.074 |
| Negative | 3 | 33% | 85 | 65% |  |
| **HBV DNA (IU/mL)** |  |  |  |  |  |
| overall | 9 | 7.3×10^6^(672,4.1×10^9^) | 134 | 750(BLQ,ALQ) | 0.005 |
| among HBeAg+ | 6 | 9.4×10^7^(9.6×10^4^, 4.1×10^9^) | 45 | 1.2×10^8^(31,ALQ) | ─ |
| among HBeAg- | 3 | 3.8×10^3^(672,2.0×10^5^) | 84 | 328(BLQ,1.3×10^6^) | ─ |
| **ALT (U/L)** |  |  |  |  |  |
| overall | 9 | 38(15,513) | 131 | 21(5,69) | 0.113 |
| among HBeAg+ | 6 | 87(15,513) | 44 | 25(12,52) | ─ |
| among HBeAg- | 3 | 17(17,39) | 82 | 21(6,69) | ─ |
| **APRI** |  |  |  |  |  |
| overall | 6 | 0.3(0.2,4.4) | 114 | 0.3(0.1,0.8) | 0.459 |
| among HBeAg+ | 4 | 0.2(0.2,4.4) | 38 | 0.3(0.1,0.7) | ─ |
| among HBeAg- | 2 | 0.4(0.4,0.4) | 73 | 0.3(0.1,0.8) | ─ |
| **Phenotype** |  |  |  |  | 0.345 |
| IT, HBeAg positive | 2 | 22 | 11 | 9 |  |
| IA, HBeAg positive | 3 | 33 | 22 | 18 |  |
| IA, HBeAg negative | 0 | 0 | 6 | 5 |  |
| IC, HBeAg negative | 1 | 11 | 36 | 29 |  |
| Indeterminant | 3 | 33 | 50 | 40 |  |

**^&^** P-value is not provided when n is too small.

*Women with non-viable outcomes or treatment-related flares were exclude.

**Figure S1. ALT -Flares: Spontaneous reactivation**

| 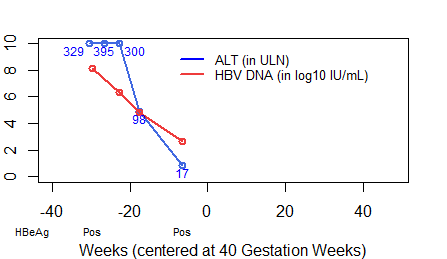 | **Subject 1**  Prior to conception: ALT 54 U/L and DNA of 1.97×10^8^ IU/ mL (8.3 log_10_ IU/mL).  **Flare:**  **Onset:** First trimester (10 weeks)  **Grade***: moderate  **Duration:** 24 weeks  **Treatment initiated:** no  **HBeAg loss:** no  **HBV DNA:**  decrease (> 10 fold) with flare |
| --- | --- |
| 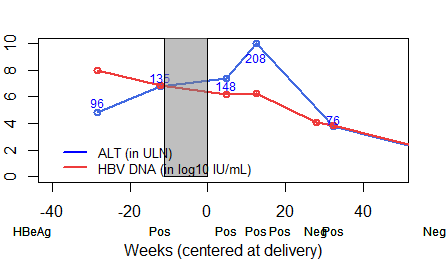 | **Subject 2**  **Flare: 1^st^ flare**  **Onset:** Second trimester (27 weeks)  **Grade**: mild  **Duration:** unknown  **Treatment**: yes  **HBeAg loss:** no  **HBV DNA:** decrease (> 10 fold) with flare |
| 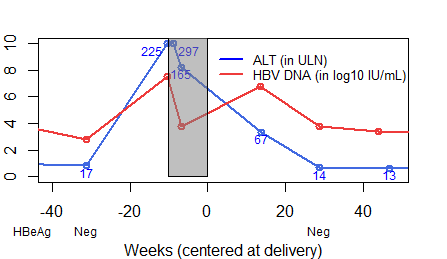 | **Subject 3**  **Flare:**  **Onset:** Third trimester (28 weeks)  **Grade***: moderate  **Duration:** 39 weeks  **Treatment initiated:** yes  **HBV DNA:** increase (> 10 fold) with flare |
| 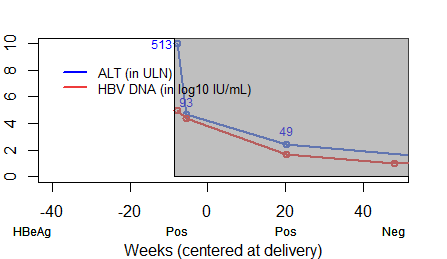 | **Subject 4**  **Flare:**  **Onset:** Third trimester (30 weeks)  **Grade***: moderate  **Duration:** >48 weeks  **Treatment initiated:** yes  **HBeAg loss:** transient  **HBV DNA change with flare:** unknown |
| 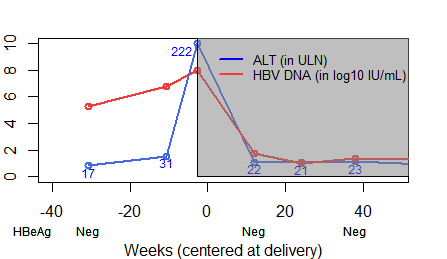 | **Subject 5**  **Flare:**  **Onset:** Third trimester (39 weeks)  **Grade****: moderate  **Duration:** 15 weeks  **Treatment initiated:** yes  **HBV DNA:** increase (> 10 fold) with flare |
| 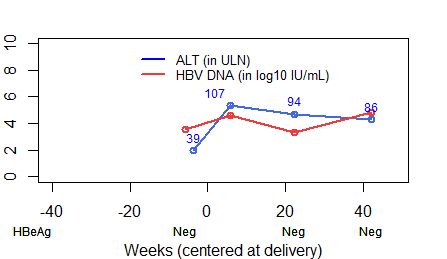 | **Subject 6**  **Flare:**  **Onset:** Post-partum (6 weeks)  **Grade**: mild  **Duration:** >36 weeks  **Treatment initiated:** no  **HBV DNA:** increase (> 10 fold) with flare |
| 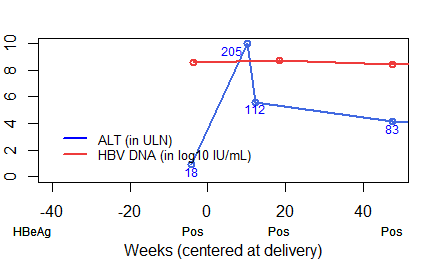 | **Subject 7**  **Flare:**  **Onset:** Post-partum (10 weeks)  **Grade****: moderate  **Duration:** >37 weeks  **Treatment initiated:** no  **HBeAg loss:** no  **HBV DNA:** no change with flare (<10 fold) |
| 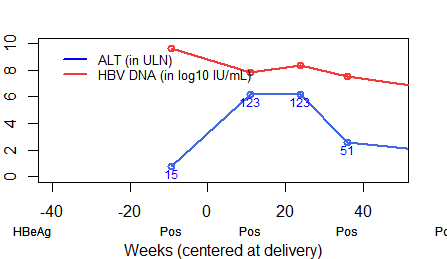 | **Subject 8**  **Flare:**  **Onset:** Post-partum (11 weeks)  **Grade**: mild  **Duration: >**25 weeks  **Treatment initiated:** no  **HBeAg loss:** no  **HBV DNA:** decrease (> 10 fold) with flare |
| 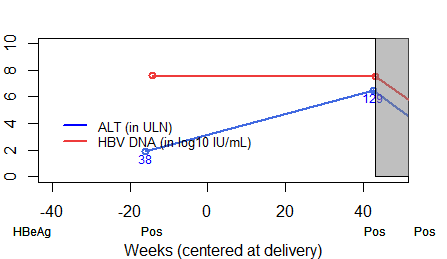 | **Subject 9**  **Flare:**  **Onset:** Post-partum (42 weeks)  **Grade:** mild  **Duration:** >5 weeks  **Treatment initiated:** yes  **HBeAg loss:** no  **HBV DNA:** no change with flare (<10 fold) |

- The blue lines represent ALT (in ULN, such that each unit represents 20 U/L). ALT values above 10 ULN (i.e., 200 U/L) were plotted at 10 ULN, with actual values (in U/L) labeled on the figure in blue.
- The red lines represent HBV DNA (in log_10_ IU/mL). For example, a value of 3 means 10^3^ IU/mL and a value of 7 means 10^7^ IU/mL. Each unit increase in log_10_ IU/mL corresponds to 10-fold increase in the original scale (IU/mL).
- The grey area means the woman was receiving antiviral treatment.
- *confirmed by HBRN Adjudication Committee to be related to pregnancy
- **unknown or not yet confirmed by HBRN Adjudication Committee to be related to pregnancy

**Figure S2. ALT Flares: on antiviral therapy**

| 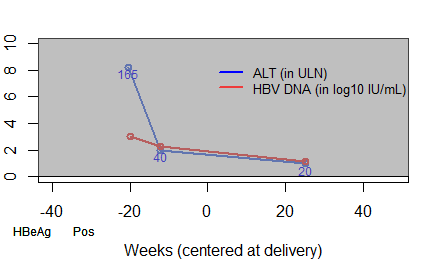 | **Subject 1**  **Flare:**  **Onset:** Second trimester (17 weeks)  **Grade:** mild  **Duration:** unknown  **HBeAg loss:** unknown  **HBV DNA:** unknown |
| --- | --- |
| 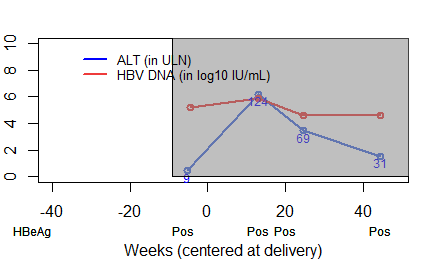 | **Subject 2**  **Flare:**  **Onset:** Post-partum (13 weeks)  **Grade**: mild  **Duration: >**31 weeks  **HBeAg loss:** no  **HBV DNA:** no change with flare (<10 fold) |
| 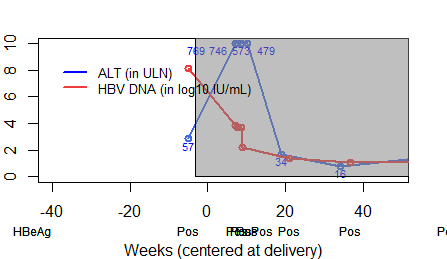 | **Subject 3**  **Flare:**  **Onset:** Post-partum (7 weeks)  **Grade***: moderate  **Duration:** 12 weeks  **HBeAg loss:** no  **HBV DNA:** decrease (> 10 fold) with flare |

- The blue lines represent ALT (in ULN, such that each unit represents 20 U/L). ALT values above 10 ULN (i.e., 200 U/L) were plotted at 10 ULN, with actual values (in U/L) labeled on the figure in blue.
- The red lines represent HBV DNA (in log_10_ IU/mL). For example, a value of 3 means 10^3^ IU/mL and a value of 7 means 10^7^ IU/mL. Each unit increase in log_10_ IU/mL corresponds to 10-fold increase in the original scale (IU/mL).
- The grey area means the woman was receiving antiviral treatment.
- *confirmed by HBRN Adjudication Committee to be related to pregnancy

**Figure S3. ALT Flares: after withdrawal of therapy**

| 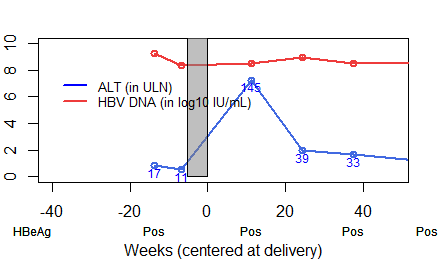 | **Subject 1**  **Flare:**  **Onset:** 11 weeks after discontinuation of therapy  **Grade**: mild  **Duration: >**26 weeks  **Retreatment**: no  **HBeAg loss:** no  **HBV DNA:** no change with flare (<10 fold) |
| --- | --- |
| 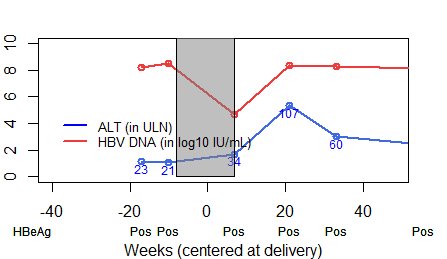 | **Subject 2**  **Flare:**  **Onset:** 14 weeks after discontinuation of therapy  **Grade**: mild  **Duration: >**12 weeks  **Retreatment**: no  **HBeAg loss:** no  **HBV DNA:** increase (>10 fold) with flare |
| 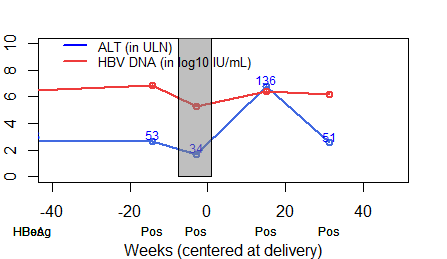 | **Subject 3**  **Flare:**  **Onset:** 14 weeks after discontinuation of therapy  **Grade**: mild  **Duration:** 16 weeks  **Retreatment**: no  **HBeAg loss:** no  **HBV DNA:** increase (>10 fold) with flare |
| 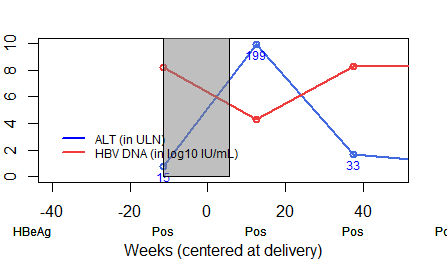 | **Subject 4**  **Flare:**  **Onset:** 7 weeks after discontinuation of therapy  **Grade**: mild  **Duration:** >25 weeks  **Retreatment**: no  **HBeAg loss:** no  **HBV DNA:** decrease (>10 fold) with flare |
| 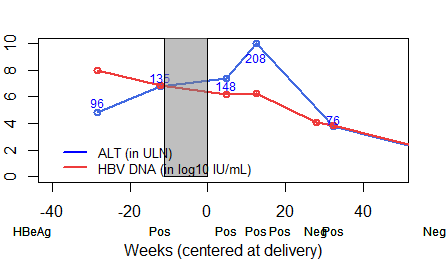 | **Subject 5**  **Flare: 2^nd^ flare**  **Onset:** 12 weeks after discontinuation of therapy  **Grade***: moderate  **Duration:** >19 weeks  **Retreatment**: no  **HBeAg loss:** Yes  **HBV DNA:** no change with flare (<10 fold) |

- The blue lines represent ALT (in ULN, such that each unit represents 20 U/L). ALT values above 10 ULN (i.e., 200 U/L) were plotted at 10 ULN, with actual values (in U/L) labeled on the figure in blue.
- The red lines represent HBV DNA (in log_10_ IU/mL). For example, a value of 3 means 10^3^ IU/mL and a value of 7 means 10^7^ IU/mL. Each unit increase in log_10_ IU/mL corresponds to 10-fold increase in the original scale (IU/mL).
- The grey area means the woman was receiving antiviral treatment.
- *confirmed by HBRN Adjudication Committee to be related to withdrawal of therapy
